# Why Some but Not Others? Exploring Factors That Determine Whether Alcohol Intoxication Increases Sexual Risk Behavior Among Men Who Have Sex with Men

**DOI:** 10.21203/rs.3.rs-7643273/v1

**Published:** 2025-10-31

**Authors:** Neil Gleason, Sharon S. Wang, Ange Vittone, Lauren Smith, Katherine Conroy, William H. George

**Affiliations:** University of Southern California; University of Washington; University of Washington; University of Washington; University of Washington; University of Washington

**Keywords:** Alcohol, Sexual Risk, HIV, Men Who Have Sex With Men, Qualitative

## Abstract

**Background:**

Experimental research demonstrates that alcohol intoxication can increase likelihood of condomless anal sex (CAS) among men who have sex with men (MSM), and qualitative research indicates MSM generally perceive this to be true. However, event-level quantitative research suggests alcohol does not affect likelihood of CAS for all MSM in all circumstances, and factors that may moderate this relationship have yet to be explored with qualitative methods.

**Methods:**

The current study sought to explore such moderating factors by conducting qualitative interviews with *N*=26 MSM who reported frequent casual sex and alcohol use. Interview transcripts were coded using thematic analysis.

**Results:**

Two main themes were identified. First, participants who perceived alcohol to affect sexual risk (*n*=11) identified many behaviors that alcohol affected including CAS, communication with partners, and partner selection. Second, a major difference between participants who reported alcohol effects and those who did not was perception of sexually transmitted infection (STI) risk. Participants who perceived alcohol to affect sexual risk generally expressed concern about STI risk and indicated that alcohol primarily affects sexual risk decisions with new sexual partners because these partners are perceived as riskier for STIs. Participants that perceived no alcohol effects (*n*=15) generally perceived minimal STI risk associated with their sexual behavior and would therefore engage in the same sexual behavior whether they were drunk or sober.

**Conclusions:**

The results of the present study suggest future research on alcohol use and sexual risk should focus on behaviors beyond CAS and should explore moderating factors like partner familiarity and perceived STI risk.

## Introduction

The ongoing global Human Immunodeficiency Virus (HIV) epidemic has disproportionately impacted men who have sex with men (MSM), with MSM accounting for 70% of new HIV diagnoses in the United States in 2021.^1^ The primary mode of HIV transmission for MSM is condomless anal sex (CAS), and while past efforts to increase condom use among MSM have been successful, condom use has diminished in recent years.^2^ This is true even among those who do not use other HIV prevention methods such as pre-exposure prophylaxis (PrEP)^3^ and it therefore remains important to understand factors that contribute to CAS in this population, such as alcohol and substance use.

There is robust experimental evidence for alcohol’s direct causal effect on increased likelihood of CAS in MSM.^4,5^ However, event-level research reports mixed findings, with some studies indicating alcohol increases likelihood of CAS, and other studies finding no effects.^6^ This indicates that alcohol may increase likelihood of CAS, but not for all individuals in all circumstances. It is therefore especially important to understand the factors that may moderate the effect of alcohol on sexual risk behaviors. Thus, at this stage in the research, qualitative methods are well suited to identify such moderating mechanisms for further study.

### Qualitative Research on Alcohol and Sexual Risk Behavior among MSM

Qualitative studies with MSM have found that MSM perceive alcohol intoxication to increase their likelihood of engaging in CAS,^7–12^ and participants in two studies specifically cited alcohol’s disinhibiting effects as a potential causal link.^8,12^ While some studies included participants who reported no or limited perceived effect of alcohol on CAS, these participants were always in the minority of their respective samples.^8,13,14^ Unfortunately, these studies have offered little insight into why some participants report alcohol effects while others do not.

Qualitative research has also not yet explored the impact of alcohol on sexual risk behaviors beyond CAS. While quantitative studies have found positive associations between alcohol intoxication and increased willingness to have anal sex^15^ and decreased use of condom negation skills,^16^ these relationships have not been explored in qualitative studies. There may be ways in which alcohol affects sexual risk behavior that is not adequately captured by the quantitative literature, and qualitative research could help identify these missing links.

### Alcohol Myopia and Inhibition Conflict

**Figure 1.**
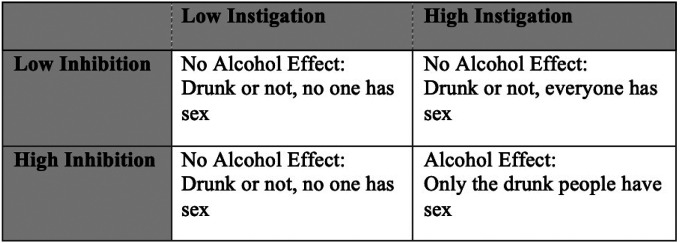
Depiction of the Inhibition Conflict Model (ICM)

The Inhibition Conflict Model (ICM) of alcohol myopia^17^ provides a useful framework for understanding when and for whom alcohol may increase likelihood of CAS. The ICM is a specified model of Alcohol Myopia Theory^18^ which postulates that alcohol intoxication narrows attentional focus toward impelling cues (i.e., instigation or “go” signals) and away from inhibiting cues (i.e., “stop” signals) for a variety of risk behaviors including sexual risk.^19^ The ICM suggests alcohol has the greatest effect on sexual risk behavior when a person is conflicted due to simultaneous high instigation and high inhibition (see Figure 1). Instigation factors are dispositional or situational factors that elevate motivation to engage in sex, such as sexual arousal, while inhibition factors are dispositional or situational factors that diminish motivation to engage in sex, such as situational danger or concerns about negative consequences^18^. If both instigation and inhibition are high, alcohol myopia would lead the person to ignore the inhibiting cues (e.g., potential negative consequences of CAS) while selectively attending to instigating cues (e.g., sexual arousal), which would then increase likelihood of engaging in a sexual risk behavior such as CAS.

### Current Study

Guided by Alcohol Myopia Theory and the Inhibition Conflict Model, the present study sought to identify the moderating impact of inhibition factors on the relationship between alcohol intoxication and sexual risk behavior among MSM using a qualitative interview design. This study takes a combined deductive and inductive approach to thematic analysis,^20^ searching for themes described in previous research while allowing for the identification of new themes emerging from the data. The research questions guiding analyses were: 1) in what ways do participants perceive alcohol to have an impact on sexual behaviors relevant to HIV and STI risk (i.e., condom use, partner choice, etc.); 2) what inhibition factors does alcohol diminish; 3) in what ways do participants perceive alcohol to have no significant impact on their sexual behavior; and 4) what are the individual and situational factors that determine whether alcohol has an impact on sexual behavior?

## Methods

### Participants and Recruitment

Participants were recruited from a larger online survey study examining HIV risk and substance use in MSM. Those who completed an online survey and indicated they were interested in participating in an interview were contacted via email or text message and invited to complete a 50-minute qualitative interview. Participants with diverse demographic characteristics (i.e., race, age, sexual identity, HIV status, PrEP use) who reported frequent sexual behavior, heavy alcohol use, and recent condomless sexual activity were purposively recruited. A total of N=65 participants were invited to complete an interview, and of these participants, N=26 completed interviews. Participant pseudonyms and interview characteristics are displayed in Table 1, and descriptive statistics of demographic factors are displayed in Table 2. While participants who reported use of drugs other than alcohol during sex were excluded from recruitment efforts (i.e., to better assess alcohol-specific effects), four participants who regularly used methamphetamine with sex were ultimately interviewed (see Table 1).

### Procedure and Measures

Ethical approval for the study was obtained from the [blinded for review] IRB. Participants were sent an online informed consent form to complete prior to the interview. Interviews were semi-structured and were conducted via phone or video call with the study PI. All interviews were conducted in English, and interviews lasted for approximately 50–80 minutes.

Interviews began with a review of informed consent, an overview of the topics that would be covered, and an opportunity for participants to address any questions or concerns. After confirming demographic information they provided in the survey (i.e., age, racial/ethnic identity, sexual orientation, etc.), participants were asked to describe their use of alcohol and other substances, including the typical frequency and settings of use, as well as any associated problems. Next, participants were asked about their partnered sexual behaviors and their perceptions of STI risk for these behaviors and their use of HIV prevention strategies (i.e., condom use, PrEP, antiretroviral therapy). Participants were then asked about how alcohol and other substance use relates to their sexual behavior, specifically probing for perceived effects of alcohol on condom use and other risk mitigation strategies.

### Analyses

#### Analysis Team Positionality

The PI identifies as a white, gay, cisgender man and is a postdoctoral research associate in psychology with vested research interests in the sexual and mental health of sexual minority individuals. In particular, he takes a sex-positive approach to treating health concerns related to sexual behavior and advocates for safe and healthy sexual enjoyment, utilizing harm-reduction strategies to approach behavior change with high-risk substance use and sexual behaviors. Other study team members that transcribed and coded the data include psychology graduate students who study sexual behavior and substance use and undergraduate and non-student volunteer research assistants.

#### Interview Transcription

Initial transcription of interviews was automated using transcription software and transcripts were reviewed, formatted, and corrected by at least two research assistants and the PI based on the original recording. Identifying information including names and geographic locations were removed from transcripts to preserve anonymity.

#### Codebook Development and Coding

The codebook was developed using both deductive and inductive approaches. First, relevant categories of codes were developed based on prior research and the ICM (i.e., “alcohol affects inhibition factors”). After completing the first 9 interviews, the study PI revised the preliminary codebook by revising existing codes and adding codes that emerged from the interviews. Gaps in the existing sample and data (i.e., lack of participants living with HIV, lack of perspective on how alcohol affects CAS for HIV-negative individuals not using PrEP) were identified and guided purposive recruitment of new interview participants. After completing all interviews, the study PI again reviewed summaries of interviews and interview notes to identify additional categories of codes.

Interview transcripts were coded using Atlas.ti (version 24). All interviews were coded by the study PI, with approximately 30% of interviews (*n*=8) being co-coded by research assistants. Research assistants first received training on the codebook and coded two interviews independently. The coding team met to compare codes and provide feedback, and research assistants then coded three additional interviews each. In team meetings, the PI and research assistants reviewed all discrepancies in codes and discussed potential changes to coding or the codebook to address these discrepancies.

#### Identification of Themes

Analyses of codes were conducted using Atlas.ti (version 24). Queries were run to consolidate quotations for each code, and co-occurrence analyses were conducted to identify codes that had significant overlap. Themes were identified across codes that helped to address the study’s primary research questions. To better clarify themes, participants were grouped based on whether or not they reported effects of alcohol on their sexual risk behavior. Participants who reported that alcohol affected their sexual behavior during a previous period of life (e.g., in college, or before they became HIV-positive), or that they could hypothetically see alcohol having an effect but did not currently report effects of alcohol on their sexual behavior, were placed into the “does not affect” group because the interview focused on their current behavior. These groups were then examined separately to identify contrasting themes

## Results

### Participant Demographics and Descriptives

A total of *N*=26 participants completed interviews and were included in analyses. Just over half of participants identified as White (N=14; 53.8%), with the remainder representing a variety of racial identities. Over half of participants reported they were HIV-negative and currently use PrEP (*n*=15; 57.7%), while *n*=5 participants (19.2%) reported that they were HIV-positive (see Table 2). After grouping participants based on alcohol effects, *n*=11 participants were placed in the “alcohol affects sexual risk behavior” group and *n*=15 participants were placed in the “alcohol does not affect sexual risk behavior” group. Pseudonyms of participants included in each group can be viewed in Table 3.

### Theme 1: Alcohol can Affect Several Sexual Risk Behaviors Beyond CAS

Eleven out of 25 participants reported that they perceived alcohol to affect their sexual risk behavior. Some of these participants described how alcohol affects their “heat of the moment” decision making during a sexual encounter.

“If we’re doing it [while intoxicated] and we’re in the living room, I’m not going to the bedroom to get [a condom]. […]I just don’t see a point. I’m like, ‘well, if I do it now, I can just go get tested in a few days and see.’ […] I’m not thinking about the future. I’m thinking about the now.”

(Jack, 24, bisexual, white, daily PrEP user)

Others described how alcohol made them more willing to agree to condomless sex *before* a sexual encounter or made them less likely to have a condom available for a sexual encounter.

“I was just rejected over and over again [because I would only have sex with condoms] and was getting very upset and just, you know, low self-esteem and all that. So I started getting really drunk and was like, ‘fuck it’. Let’s do it [i.e., have sex without a condom].”

(Jordan, 30, gay, white, 2-1-1 PrEP user)

“I’m more prone to be adamant about condom usage if I’m not drinking. Because I’m probably not like heated or aroused as much as I would be if I’ve been drinking. […] Sober, I’m gonna ask, whether texting or in person, ‘are you okay with condoms? Do you have a condom?’ If they don’t have a condom, I’ll say, ‘I’ll bring them.’”

(Jerome, 33, gay, Black, daily PrEP user)

Several participants described no effects of alcohol on their condom use (largely because they do not use condoms in general) but described how alcohol made them more likely to have anal sex with a sexual partner rather than engaging in lower-risk behaviors like oral sex or mutual masturbation.

“When it comes to what kind of sex to have, like in terms of just oral versus going over to anal, I would say that there’s probably a small impact that alcohol would have in terms of nudging it further. […] Because if I’ve decided to do one thing and I consume a little bit of alcohol, then probably I’ll be suggestible to doing more.”

(Josh, 28, gay, white, daily PrEP user)

Many participants also described how alcohol intoxication affected the screening of new sex partners. For instance, participants described how when they are intoxicated, they are less likely to ask about STI status and PrEP use.

“On Halloween [when I was drunk] I had a guy come over and it wasn’t until the morning after - I woke up and I’m like, ‘wait, I just got his pictures. I didn’t even see his status or even ask him for his status.’ I’m like, ‘Crap. Why didn’t I check?’”

(Diego, 36, gay, Latino, daily PrEP user)

Other participants described how when they were intoxicated, they were more likely to seek out new sexual partners or to have sex on a night when they were not originally intending to do so.

“I’m not gonna be like, ‘oh, I’m gonna drink and hook up with someone tonight.’ It’s more so I drink and then I’m in a situation that ends up leading into that.”

(Nate, 24, bisexual, Latino, daily PrEP user)

Finally, one participant discussed how alcohol affected his ability to use PrEP. Keith, who up until a few weeks prior to the interview was struggling with a severe alcohol use disorder, described how his alcohol use prevented him from adhering to daily PrEP.

“When I was drinking I couldn’t remember to take a pill every day. It just seemed like I was wasting my insurance money on getting it cause I would forget half the days if I was drunk. […] They brought up [long-acting PrEP] at the [sexual health] clinic, but my insurance doesn’t cover it.”

(Keith, 38, bisexual, white, no PrEP use)

Taken together, the experiences of these participants highlight how alcohol can affect a variety of sexual risk decisions. These decisions include decisions related to condom use, like whether to use a condom, ensure a condom is available, and communicate their preferences to a partner. However, participants also described decisions unrelated to condom use, such as the decision to have sex with a partner perceived to be risky, have anal sex with a partner rather than oral sex or mutual masturbation, screen a partner for risk factors, whether to look for a partner in the first place, and whether to initiate PrEP use. These decisions happen during the sexual encounter itself, but also happen immediately before the sexual encounter or, in the case of PrEP use, days or weeks before.

### Theme 2: Alcohol Does Not Affect Sexual Behavior When STI Risk is Perceived to be Low

The primary factor distinguishing those reporting effects of alcohol on sexual risk behavior (N=12) and those reporting no effects (N=14) was perception of STI risk. Concerns about STIs were reported by all 12 participants who reported alcohol effects and only four participants who reported no effects. Representative quotations from participants in each group about their perceptions of STI risk and the effects of alcohol on these perceptions are displayed in Table 3.

The 12 participants who reported alcohol effects all perceived significant STI risk and were concerned about potential negative consequences such as painful symptoms, long-term consequences, or treatment side effects. These concerns were based on firsthand experiences, secondhand experiences of peers, and sexual education they received growing up. Several participants in this group were also very concerned about the stigma associated with STIs. Some were concerned about how others, such as romantic partners, sexual partners, or peers, would judge them if they tested positive for an STI. Others discussed negative consequences of past STI diagnoses, such as feeling “dirty” and shameful or experiencing of rejection from others.

The 14 participants who reported no alcohol effects generally described a lack of perceived STI risk. Most felt they were sufficiently protected from HIV, either by using PrEP or maintaining an undetectable viral load and saw other STIs as easily detectable and treatable. Even participants who were not currently protected from HIV with PrEP or use of anti-retroviral therapy (ART) stated they were not concerned about STIs, noting they had not personally experienced negative consequences of STIs or had not been told by health educators or providers about the risks STIs posed. They therefore perceived (perhaps inaccurately) that they were at low risk for STIs and that their existing strategies for avoiding STIs were sufficient. Three of the 15 participants in this group did report some concern about STIs, but these participants expressed more ambivalence about STI risk compared to those who reported alcohol effects.

“I had quite a complication from syphilis last year where it amassed into a tumor in my rectum, which really caused a lot of health issues. […] [STIs are] a worry, but then… it does come with the territory of being gay just having sex in general and not using protection. And of course the [city] hookup culture is like, if you put in ‘safe’ [on your hookup app profile] it’s like, ‘blocked’ right away.”

(Brian, 34, gay, white, daily PrEP user)

These participants, like Brian, expressed concern about STI risk but also felt the risk was inherent to being sexually active. Thus, these participants may have lacked sufficient inhibition (i.e., STI risk concerns) for there to be noticeable effects of alcohol effects.

Of note, nine participants described how they perceive new sexual partners (i.e., hookups) to be higher risk for STIs compared to established sex partners (i.e., partners they have hooked up with before, casual partners, “friends with benefits,” etc.). They were therefore more likely to use condoms or refrain from anal sex with these partners, reserving unprotected sex for partners they had met previously or established a friendship with. One participant elaborated on how alcohol differentially affected his sexual risk behavior with new and established partners.

“If I know you or if you’re a consistent person and I know like you’re getting tested, you’re being safe, then I’m less likely to use [a condom]. Now if it’s a one-off situation, it really depends on if we were drinking and how drunk I got before. I will be like, okay, ‘I should wear one’ or ‘too buzzed, just screw it.’”

(Jack, 24, white, bisexual, daily PrEP user)

This highlights the perception that because sex with a new partner is perceived as riskier and is more likely to involve condoms and other risk-mitigating behaviors, there are more inhibitions for alcohol to disinhibit and more sexual risk behaviors for alcohol to enable in these encounters.

It is also important to note that while these participants *perceived* established partners to be lower risk than hookup partners, these perceptions were often based on assumptions and gut feelings.

I just feel more comfortable with somebody I already know. Like, you don’t have it [HIV] now unless you fucking with new people… you probably are, but I would assume you’re taking some type of precaution”

(Charles, 29, bisexual, Black HIV- and not using PrEP)

“When you talk to someone sometimes you can get a sense of if they’re someone that maybe hooks up 3 times a year versus someone that is looking to hook up with different people every day [..] Sometimes you have that gut feeling. You’ll just be like, okay, maybe I shouldn’t hook up with this person.”

(Todd, 23, Asian, gay, long-acting PrEP user)

One participant expressed self-awareness of how his perceptions of risk may be inaccurate.

“I suppose like so many people, I’m dumb enough to really think I could tell if someone might be carrying an STI or not.”

(Ian, 67, gay, white, HIV+ and undetectable)

This participant’s blunt assessment highlights how perceived risk for STIs with established partners likely differ from actual risks, and how these misperceptions can lead to risker behavior.

## Discussion

Analyses of these qualitative interviews provided a rich description of how MSM perceive alcohol to affect or not affect their decision making around sexual risk. A key takeaway from the first theme is the need to explore the effects of alcohol sexual risk behavior beyond condom use during sexual encounters, which has largely been the focus of experimental and event-level research up to this point.^19^ Participants described how alcohol not only affected sexual decisions the “heat of the moment” during a sexual encounter, but also affected several decisions leading up to the sexual encounter, such as making sure a condom is available and deciding whether have meet up with a partner who does not want to use a condom. Participants also described many decisions other than condom use that were affected by alcohol intoxication, such as the decision to have anal sex, screening a partner for risk factors, and choosing to seek out a partner in the first place. These findings aligns with previous event-level and experimental research indicating alcohol intoxication increases likelihood of having sex with a potential partner^21,22^ and having anal sex.^15,23^ However, this is the first qualitative study to explore the variety of behaviors alcohol can affect in MSM. These results highlight that regardless of whether alcohol affects someone’s decision to use a condom in the heat-of-the-moment, it may affect their sexual risk in other ways before the sexual encounter happens.

The second theme highlighted the importance of perceived STI risk in determining whether alcohol has an effect on sexual behavior, which is consistent with the ICM. Participants described how alcohol intoxication causes them to attend to sexual excitation cues (i.e., sexual arousal) rather than inhibition cues (i.e., risk of STIs), and only participants with salient inhibition cues (i.e., who perceived STI risk) reported alcohol effects. These results suggest that STI risk perception is a particularly important inhibition factor for CAS in MSM and may moderate the association between alcohol intoxication and CAS. In addition, this theme confirms prior research indicating that sexual behavior with new partners is more likely to be affected by alcohol than sexual behavior with established partners,^24^ and indicates that this difference is likely due to perceived STI risk. However, these results also highlight that greater care is needed in the assessment of partner familiarity. Many event-level studies have distinguished between romantic partners and casual partners, but did not distinguish further, for instance, assessing the number of times they had been sexual with a casual partner.^15,21,24,25^ Given that some participants noted increased comfort with a partner after one or two encounters, it may be important to investigate finer-tuned distinctions on the spectrum of partner familiarity.

Finally, it is notable that while many participants perceived lower STI risk with established partners compared to newer partners, these perceptions were often based on potentially inaccurate assumptions. This is in line with previous research indicating MSM tend to perceive sex with regular partners as lower risk for STIs, and therefore are less likely to use condoms.^26^ It also aligns with research indicating perceptions of STI risk with regular partners tend to be based on unreliable indicators of risk, including attractiveness and emotional safety.^27^ These findings suggest that alcohol-focused STI risk interventions should help individuals to re-evaluate how they determine STI risk, helping them to establish more effective tools for risk assessment.

## Limitations

Several limitations must be accounted for when interpreting these results. First, the sample for this study was small and was purposively recruited to include only MSM at high risk for STIs who drink alcohol with sex. Recruitment for this study was limited to individuals who use hookup applications or who are members of crowdsourcing platforms, and there were relatively few participants who did not use PrEP or had an unknown viral load. These factors limit the generalizability of this study’s findings, as the participants are not representative of the larger MSM population, especially those at highest risk for HIV (i.e., those not consistently using PrEP or ART). However, in-depth, exploratory nature of these qualitative interviews trades generalizability with internal validity and provides an opportunity to uncover factors that have been missing from quantitative research up to this point.

Second, this study explored participants’ perceptions of alcohol’s effects on their own behavior, and these perceptions are subject to bias, such as hindsight bias and social desirability bias. Thus, participants may have attributed certain decisions to alcohol intoxication when these decisions were actually caused by other factors. On the other hand, participants may have downplayed alcohol’s effects or misunderstood the effects alcohol had on them in the moment. Despite this limitation, research on alcohol expectancies has shown that individuals’ perceptions of alcohols’ effects on their behavior has a strong influence on their behavior when drinking, even when they are given a placebo non-alcoholic drink in an experimental setting.^28^ It is therefore important to understand perceptions of alcohol’s effects, given that these perceptions will likely have real influence above and beyond the physiological effects of alcohol.

## Future Research

Results of this study suggested that perceived STI risk is a key factor in determining whether alcohol has an impact on sexual risk behavior. Future quantitative research should therefore attempt to confirm this finding by comprehensively assessing perceptions of STI risk and including this variable as a moderator. Such research could help to resolve the discrepancies between experimental and event-level research. However, research may first be needed to properly operationalize the measurement of STI risk perception, as it is likely a heterogeneous construct. Participants in this study had diverse concerns about STIs, including concerns related to both physical health and social stigma, and many participants expressed ambivalence about STI risk. Assessment that captures all aspects of STI risk perception, both at the personality and event-level, must be developed in order to determine the moderating effects of this factor.

Additionally, future quantitative research should also explore ways to more precisely measure partner familiarity among MSM. Like STI risk, this construct is multifaceted and may involve both number of prior encounters as well as perceptions of trust and safety with the partner. Partner familiarity may serve as another important moderating variable in the relationship between alcohol and sexual risk, and thus establishing a clear definition and a valid measurement of this construct is paramount for future research.

## Conclusions

This study sought to explore effects of alcohol on sexual risk behavior and factors that may moderate this relationship among MSM. Results confirmed the inhibition conflict model as an important framework for understanding alcohol’s effects, and highlighted perceptions of STI risk and partner familiarity as key potential moderating factors. Despite limitations in generalizability, this study points to future directions in both qualitative and quantitative research, emphasizing the need for valid measurement and more precise recruitment of high-risk populations.

## Figures and Tables

**Table 1:** Data Overview

Participant Pseudonym	Age	Sexual Identity	Race	HIV Status/PrEP use	Interview Date	Length (minutes)	Other Notes
Carlos	24	Bisexual	Latino	Daily PrEP	9/12/23	50	
Brian	34	Gay	White	Daily PrEP	9/15/23	54	
Nate	24	Bisexual	Latino	Daily PrEP	9/18/23	51	
Daniel	33	Gay	White	HIV- No PrEP	9/19/23	46	Reported severe alcohol use disorder
Jack	24	Bisexual	White	Daily PrEP	9/19/23	46	
Josh	28	Gay	White	Daily PrEP	9/22/23	65	
Todd	23	Gay	Asian	Long-acting PrEP	9/26/23	73	
Eric	51	Gay	Latino	Daily PrEP	9/30/23	41	
Keith	38	Bisexual	White	HIV- No PrEP	10/4/23	48	Reported severe alcohol use disorder
Thomas	31	Queer	Black	2-1-1 PrEP	10/10/23	61	
Alvaro	30	Gay	Latino	2-1-1 PrEP	10/11/23	53	
David	55	Gay	White	HIV+ and undetectable	10/17/23	67	Reported current meth use
Charles	29	Bisexual	Black	HIV- No PrEP	10/24/23	49	
Will	37	Gay	White	Long-acting PrEP	10/24/23	56	Reported past meth use
Jordan	30	Gay	White	2-1-1 PrEP	10/25/23	51	
Jerome	33	Gay	Black	Daily PrEP	11/8/23	55	
Fred	43	Bisexual	Multiracial	HIV- No PrEP	11/10/23	44	Reported current meth use
Diego	36	Gay	Latino	Daily PrEP	11/10/23	59	
James	41	Gay	White	2-1-1 PrEP	11/14/23	63	
Antonio	31	Gay	Latino	Daily PrEP	11/14/23	64	
Paul	35	Gay/Queer	Latino	Daily PrEP	11/15/23	56	
Juan	24	Gay	Latino	Daily PrEP	11/15/23	54	
Chris	48	Gay	White	HIV+ undetectable	11/15/23	60	
Adam	32	Gay	White	HIV+ unknown if undetectable	11/21/23	55	Reported current meth use
Ian	67	Gay	White	HIV+ undetectable	11/28/23	87	
Ryan	39	Gay	White	HIV+ undetectable	11/28/23	59	Reported current meth use

**Table 2.** Demographic Characteristics and Recruitment Sources of Sample

Demographic Factor	N (%)
Age	M=35.46; SD=10.48
	Min=23; Max =67
Sexual Orientation	
Gay	18 (69.2%)
Bisexual	6 (23.1%)
Queer/Pansexual	2 (7.7%)
HIV Status	
HIV-	20 (76.9%)
Not on PrEP	4 (20%)
Daily PrEP	11 (55%)
On-Demand PrEP (2-1-1)	3 (15%)
Long-acting PrEP	1 (10%)
HIV+	5 (19.2%)
Undetectable	4 (80%)
Inconsistent med use	1 (20%)
Unknown HIV Status	1 (3.8%)
Race/Ethnicity	
White (non-Hispanic)	13 (50.0%)
Hispanic/Latino	8 (30.8%)
Black/African American	3 (11.5%)
Asian	1 (3.8%)
Multiracial	1 (3.8%)
Relationship Status	
Single	19 (73.1%)
Non-monogamous relationship	7 (26.9%)
US Region	
Northeast	2 (7.7%)
Mid-Atlantic	2 (7.7%)
Southeast	4 (15.4%)
Midwest	4 (15.4%)
Southwest	5 (19.2%)
West	9 (34.6%)
Religious affiliation	
Christian	10 (38.5%)
Buddhist	1 (3.8%)
Atheist/Agnostic/None	14 (53.8%)
No response	1 (3.8%)
Recruitment Source	
Sniffles	17 (65.4%)
Grindr	4 (15.4%)
Hornet	1 (3.8%)
Prolific	4 (15.4%)

**Table 3.** Group Differences Between Participants that Did and Did Not Report Alcohol Effects on Sexual Risk Behavior

	Participant Groups	
	Alcohol Affects Sexual Risk Behavior (N=12)	Alcohol Does Not Affect Sexual Risk Behavior (N=14)
Participants	Nate, Jack, Todd, Keith, Will, Jordan, Jerome, Diego, James, Antonio, Paul, Juan	Carlos, Brian, Daniel, Josh, Eric, Thomas, Alvaro, David, Charles, Fred, Chris, Adam, Ian, Ryan
Perceptions of STI risk	*“I have extreme anxiety over any STI. I was talking to somebody who had had syphilis and he said while it was able to be cured, the needle in his ass was very painful.”* (Jordan, 30, gay, white, 2-1-1 PrEP user) *“I personally hate, hate, hate, hate, hate it when I get [an STI] You have to go get treatment, and treatment can hurt depending on which one it is.”* (Nate, 24, bisexual, Latino, daily PrEP user) *“The very first time that I got an STI was a few years ago and I was dating somebody. That person didn’t take this news well, and me getting an STI actually ended the relationship. So now I have that on my mind.”* (Diego, 36, gay, Latino, daily PrEP user)	*“[Regarding testing positive for an STIjYou know, if it happens it happens and there’s treatment. […] With the partners I have, it’s very much this understanding that we know what we’re getting into and if it should happen [that we test positive] then we Just tell each other and move on.”* (Chris, 48, gay, white, HIV+ and undetectable) *“[STIs] worry me, but obviously not enough for me to use a condom. […] I’ve never had another STI [other than HIV]”* (Ian, 67, gay, white, HIV+ and undetectable) *Not once has any [worry about STIs] even crossed my mind. I never had any STIs and I’m the type of person that… I have to go through something, I have to learn the hard way […] I’ve really never had doctors or anybody in my adult life Just talk to me about it [STIs].”* (Adam, 32, gay, white, HIV+, unknown if undetectable)
Effects of alcohol on perceptions of STI risk	*“[When I’m drunk] the part of me that’s more concerned with my safety and not catching STIs Just goes out the window and* I *Just do what feels good at the moment.”* (Juan, 24, gay, Latino, daily PrEP user) *“Sober I know, like intellectually, I need to worry about [STIs]. I need to make sure I don’t catch this thing that could possibly kill me, or I could give it to somebody else. But when I’m drinking, that Just all goes out the window. It Just doesn’t even cross my mind. […] Like performing well and enjoying [sex] is more important than anything else.”* (Keith, 38, bisexual, white, no PrEP use)	*“If someone wants to use a condom, they can still use a condom. […] I know what I like and the alcohol’s not going to change what I like. I’m not being convinced by alcohol to do something that I wouldn’t normally do.”* (Chris, 48, gay, white, HIV+ and undetectable) *“In terms of condom use, [alcohol] has no effect. I don’t really bother with condoms anyway. I don’t really find that I care enough to use one so alcohol doesn’t impact that one way or another.”* (Josh, 28, gay, white, daily PrEP user)

## Data Availability

The datasets generated and/or analyzed during the current study are not publicly available due to protection of participant privacy.

## Abbreviations

MSM: Men who have sex with men
CAS: Condomless anal sex
HIV: Human immunodeficiency virus
PrEP: Pre-exposure prophylaxis
ART: Anti-retroviral therapy
STI: Sexually transmitted infection
ICM: Inhibition conflict model
